# Investigation of TLR4 Antagonists for Prevention of Intestinal Inflammation

**DOI:** 10.1007/s10753-022-01714-0

**Published:** 2022-07-22

**Authors:** Janine S. Y. Tam, Janet K. Coller, Clive A. Prestidge, Joanne M. Bowen

**Affiliations:** 1 Discipline of Physiology, School of Biomedicine, University of Adelaide, Adelaide, South Australia 5005 Australia; 2 Discipline of Pharmacology, School of Biomedicine, University of Adelaide, Adelaide, South Australia Australia; 3 Clinical and Health Sciences, University of South Australia, Adelaide, South Australia Australia; 4 ARC Centre of Excellence in Convergent Bio-Nano Science and Technology, Adelaide, South Australia Australia

**Keywords:** toll-like receptor 4 (TLR4), TLR4 antagonist, intestinal inflammation, intestinal mucositis, SN-38

## Abstract

Activation of toll-like receptor 4 (TLR4) has been shown to be a major influence on the inflammatory signalling pathways in intestinal mucositis (IM), as demonstrated by TLR4 knock-out mice. Pharmacological TLR4 inhibition has thus been postulated as a potential new therapeutic approach for the treatment of IM but specific TLR4 inhibitors have yet to be investigated. As such, we aimed to determine whether direct TLR4 antagonism prevents inflammation in pre-clinical experimental models of IM. The non-competitive and competitive TLR4 inhibitors, TAK-242 (10 µM) and IAXO-102 (10 µM), respectively, or vehicle were added to human T84, HT-29, and U937 cell lines and mouse colonic explants 1 h before the addition of lipopolysaccharide (LPS) (*in vitro*: 100 µg/mL; *ex vivo*: 10 µg/mL), SN-38 (*in vitro*: 1 µM or 1 nM; *ex vivo*: 2 µM), and/or tumour necrosis factor-alpha (TNF-α) (5 µg/mL). Supernatant was collected for human IL-8 and mouse IL-6 enzyme-linked immunosorbent assays (ELISAs), as a measure of inflammatory signalling. Cell viability was measured using XTT assays. Explant tissue was used in histopathological and RT-PCR analysis for genes of interest: TLR4, MD2, CD14, MyD88, IL-6, IL-6R, CXCL2, CXCR1, CXCR2. SN-38 increased cytostasis compared to vehicle (*P* < 0.0001). However, this was not prevented by either antagonist (*P* > 0.05) in any of the 3 cell lines. Quantitative histological assessment scores showed no differences between vehicle and treatment groups (*P* > 0.05). There were no differences in *in vitro* IL-8 (*P* > 0.05, in all 3 cells lines) and *ex vivo* IL-6 (*P* > 0.05) concentrations between vehicle and treatment groups. Transcript expression of all genes was similar across vehicle and treatment groups (*P* > 0.05). TLR4 antagonism using specific inhibitors TAK-242 and IAXO-102 was not effective at blocking IM in these pre-clinical models of mucositis. This work indicates that specific epithelial inhibition of TLR4 with these compounds is insufficient to manage mucositis-related inflammation. Rather, TLR4 signalling through immune cells may be a more important target to prevent IM.

## INTRODUCTION

Toll-like receptors (TLRs) are an important class of pattern recognition receptors of the innate immune system and are expressed on a variety of both immune cells (macrophages, dendritic cells) and non-immune cells (epithelial cells) in the intestine [1–4]. Each TLR family member contains a ligand-specific extracellular domain and conserved intracellular domain, which allows highly selective responses to intestinal environmental stimuli, including homeostatic, pathogenic, and damage-associated signals [5, 6]. However, TLRs can also amplify immune responses under stress conditions which leads to chronic inflammation [7–11].

TLR4, the best studied TLR family member in the context of infection and inflammation is primarily beneficial to the intestine as it induces an inflammatory response to provide protection from invading bacteria and promotes mucosal integrity [12]. However, TLR4 can also be overexpressed in chronic inflammatory conditions such as inflammatory bowel disease (IBD), whereby people with ulcerative colitis (UC) have a 2.3-fold increase (*P* = 0.02) and people with Crohn’s disease (CD) have a 1.7-fold increase (*P* = 0.04), compared to people who have normal colonic mucosal tissue [13]. Signal transmission mediated by the upregulation of TLR4 promote the sustained release of pro-inflammatory cytokines [14] (e.g. interleukin-1 beta (IL-1β), IL-6, and tumour necrosis factor-alpha (TNF-α)). This, in turn, develops and persists as intestinal inflammation, and has also been associated with risk of inflammation-associated colon cancer [15, 16].

The pathobiology of acute intestinal inflammation as seen in intestinal mucositis (IM) in people with cancer following chemotherapy with irinotecan has also been linked to the activation of TLR4. In IM, TLR4 activation upregulates pro-inflammatory cytokines TNF-α and IL-6 [17]. This occurs via a downstream signalling pathway whereby chemotherapeutic agents cause direct injury to the intestinal epithelial cells, allowing the luminal antigens to enter the lamina propria. Lipopolysaccharides (LPS), or endotoxins, are a product of luminal antigens which activate TLR4 expressed on the intestinal epithelial layer and mucosa-associated immune cells when the luminal antigens cross over the damaged epithelial layer [18]. Subsequently, causing inflammation and ulceration. Ulceration then leads to enhanced translocation of luminal contents and increases the risk of bacteraemia in immunocompromised patients [18]. Previous study has shown that the genetic deletion of TLR4 renders mice resistant to chemotherapy-induced mucositis [19]. However, due to limitations of genetically modified animals in research translation, research efforts are now targeted at tailoring methods of inhibiting TLR4 pharmacologically.

Currently, TLR4 antagonists are being investigated for their potential in managing inflammatory-based diseases such as sepsis and arthritis [20, 21]. TAK-242 is a small-molecule TLR4 inhibitor that interferes with the down-stream signalling mediated by the CD14–TLR4 complex without directly inhibiting the binding of LPS to TLR4 [22]. It had previously undergone clinical trials as a treatment for severe sepsis [23], while IAXO-102 is a synthetic glycolipid that modulates TLR4 activation and signalling by interfering selectively with the TLR4 co-receptors CD14 and MD-2 [24]. IAXO-102 has only been used in experimental studies in abdominal aortic aneurysms [25]. There is a significant lack in studies using these antagonists (such as TAK-242 and IAXO-102) in IBDs such as IM. This study therefore aimed to investigate the potential of the TLR4 antagonists, TAK-242 and IAXO-102, to attenuate intestinal inflammation using *in vitro* and *ex vivo* models.

## MATERIALS AND METHODS

### Chemicals

TLR4 antagonists TAK-242 (Sapphire Bioscience, Australia) and IAXO-102 (Innaxon, UK) and TLR4 agonists and inflammatory mediators, LPS O55:B5 (Sigma-Aldrich, USA), SN-38 (Tocris Bioscience, United Kingdom) and TNF-α (Research and Diagnostic Systems, United States), were reconstituted according to manufacturer’s instructions for *in vitro* and *ex vivo* experiments: TAK-242: DMSO; IAXO-102: DMSO and ethanol; LPS: sterile MilliQ water; SN-38: DMSO; TNF-α: sterile PBS and 0.1% bovine serum albumin.

### *In Vitro* Human Cell Culture

Human colorectal adenocarcinoma cell lines T84 and HT-29 were grown in DMEM (Thermo Fisher Scientific, United States) supplemented with 10% FBS (Scientifix Pty Ltd., Australia) and 1% penicillin–streptomycin (Sigma-Aldrich) to simulate intestinal colonocytes. In contrast, the pro-monocytic, human myeloid leukaemia cell line U937 which are innate monocyte-like immune cells were grown in RPMI-1640 (Thermo Fisher Scientific) supplemented with 10% FBS (Scientifix Pty Ltd.) and 1% penicillin–streptomycin (Sigma-Aldrich). All cells were grown in 75 cm^2^ flasks in a 37 °C incubator with 5% CO_2_. For all experiments, all cells were used between passages 3 and 20.

T84, HT-29, and U937 cell viability were evaluated using a Cell Proliferation Kit II (XTT) (Merck & Co., United States). One hundred microliters of suspension containing T84: 5 × 10^4^; HT-29: 1 × 10^4^; U937: 3 × 10^4^ cells were seeded in 96-well plates (Corning, USA). The plates were then incubated for 24 h at 37 °C in 5% CO_2_. After 24 h incubation, the medium was replaced, and the cells were treated with TAK-242 (10 μM) and IAXO-102 (10 μM) and incubated at 37 °C with CO_2_ for 1 h. Cells were then treated with LPS (100 ng/mL), active metabolite of irinotecan SN-38 (T84 & HT-29: 1 µM; U937: 1 nM) and TNF-α (5 µg/mL) and incubated at 37 °C with CO_2_ for 36 h. Concentrations of all treatments used as well as incubations times were from previous [25, 26] and extensive dose finding studies. DMSO (0.1%) was used as the vehicle treatment. After the 36-h incubation period, the media was replaced with 100 µL media and 50 µL of XTT solution (composed of 5 mL XTT labelling reagent and 100 µL of electron coupling reagent). The plate was then incubated for 4 h at 37 °C with 5% CO_2_. Then, the cell viability was measured using a Synergy™ Mx Monochromator-Based Multi-Mode Microplate Reader (BioTek, Vermont, United States) at 490 nm. Percentage (%) of cell cytostasis was calculated using the following equation:

$$\mathrm{Cytostasis }\left(\mathrm{\%}\right)= \left[\frac{\mathrm{A}490\mathrm{ Vehicle}-\mathrm{A}490\mathrm{ Treated}}{\mathrm{A}490\mathrm{ Vehicle}}\right]\times 100$$

### *Ex Vivo* Culture of Mouse Colonic Explants

The study was approved by the University of Adelaide Animal Ethics Committee and complied with the National Health and Research Council Australia Code of Practice for Animal Care in Research and Training (2020). Twelve wild-type C57BL/6 mice (Animal Resource Centre, Australia) were culled via CO_2_ inhalation and cervical dislocation. The entire gastrointestinal tract was removed, and the colons were flushed with chilled 1 × phosphate buffered saline (PBS) (Thermo Fisher Scientific) to remove contents. The colon was then divided into 9 equal pieces and stored in chilled 1 × PBS. Each piece was cut longitudinally along the mesentery line, flattened onto a piece of manila paper and placed lumen side down in a 24-well plate (Corning) containing RPMI (400 µL) media and stored in an incubator at 37 °C at 5% CO_2_ for 10 m to equilibrate. Tissue was pre-treated with TAK-242 (10 μM) and IAXO-102 (10 μM) for 1 h before the administration of LPS (100 ng/mL) and SN-38 (2 µM) for 3 h at 37 °C at 5% CO_2_. After 3 h, the tissue and supernatant were collected and processed. Concentrations of all treatments used as well as incubation times were taken from the *in vitro* study. DMSO was used as the vehicle treatment. Following treatment, all explant supernatant was collected and stored at −20 °C for enzyme-linked immunosorbent assays (ELISAs). In addition, the explant tissue was either fixed in 10% neutral buffered formalin (ChemSupply Australia Pty Ltd., Australia) for 24 h, transferred to 70% ethanol (ChemSupply Australia Pty Ltd.) and embedded in paraffin wax (ChemSupply Australia Pty Ltd.) for histopathological analysis, or immediately snap-frozen in liquid nitrogen and stored at −80 °C for real-time polymerase chain reaction (RT-PCR) analysis.

### Histopathological Analysis of Distal Colonic Explant Tissue

Haematoxylin and eosin (H&E) staining was performed using 5 µm sections of the embedded explant tissue, cut on a rotary microtome and mounted onto glass microscope slides (Thermo Fisher Scientific). Slides were scanned and assessed (× 100 magnification) using a NanoZoomer 2.0-HT slide scanner (Hamamatsu Photonics, Shizuoka Pref., Japan). All slides underwent qualitative histopathological assessment to generate an injury score. The histological criteria used in the assessment were as follows: epithelial disruption; crypt loss; crypt abscesses; goblet cell loss; oedema; submucosal thickening; muscularis externa thickening; and polynuclear cell infiltration [27]. Each parameter was scored as: 0 = absent; 1 = mild; 2 = moderate; or 3 = severe, with a possible maximum score of 24.

### Immunohistochemistry Assessment of Cellular Markers of Apoptosis and Proliferation

Immunohistochemistry (IHC) was carried out on 5 μm sections of explant tissue, cut on a rotary microtome and mounted onto FLEX IHC microscope slides (Flex Plus Detection System, Dako; #K8020). IHC analysis was performed for caspase-3 (Abcam; #ab4051), a marker of apoptosis, and Ki67 (Abcam; #ab16667), a marker of proliferation. Changes in both parameters are validated markers for altered tissue kinetics and an excellent way to assess the subclinical severity of toxicity [28]. IHC analysis was performed using Dako reagents on an automated machine (AutostainerPlus, Dako; #AS480) following standard protocols supplied by the manufacturer. Briefly, sections were deparaffinised in xylene and rehydrated through graded ethanols before undergoing heat-mediated antigen retrieval using an EDTA/Tris buffer (0.37 g/L EDTA, 1.21 g/L Tris; pH 9.0). Retrieval buffer was preheated to 65 °C using the Dako PT LINK (pre-treatment module; Dako; #PT101). Slides were immersed in the buffer, and the temperature was raised to 97 °C for 20 min. After returning to 65 °C, slides were removed and placed in the Dako AutostainerPlus (Dako; #AS480) and stained following manufacturer's guidelines. Negative controls had the primary antibody omitted. Slides were scanned using the NanoZoomer (Hamamatsu Photonics) and assessed with NanoZoomer Digital Pathology software view.2 (Histalim). The criteria used in the assessment were as follows according to percentage (%) of area positively stained for either Ki67 or Caspase 3: < 25% = 0; 25% = 1; 50% = 2; 75% >  = 3.

### RT-PCR of Human Cell Culture and Colonic Explant Tissue

RNA was isolated from T84, HT-29, U937 and snap frozen colonic intestinal explant tissue using the NucleoSpin RNA Plus kit (Scientifix Pty Ltd., Victoria, Australia) following the manufacture’s protocol. RNA was quantified using a Synergy™ Mx Monochromator-Based Multi-Mode Microplate Reader (BioTek) and reverse transcribed using an iScript cDNA Synthesis Kit (Bio-Rad Laboratories, California, United States) according to the manufacturer′s protocol. cDNA was quantified using a Synergy™ Mx Monochromator-Based Multi-Mode Microplate Reader (BioTek) and diluted to a working concentration of 100 ng/μL. Primers for genes of interest were designed using web-based primer design program, PRIMER 3 (v. 0.4.0) [29, 30] and manufactured by Sigma-Aldrich (Missouri, United states). A list of all the primers used is shown in Table 1. Amplified transcripts were detected by SYBR Green (Quantitect, Qiagen Pty Ltd., Victoria, Australia) in a Rotor-Gene Q Series Rotary Cycler (Qiagen Pty Ltd., Victoria, Australia). All reactions were completed in triplicate. Fold change in mRNA expression was calculated using the 2^(delta CT)^ (2^∆Ct^) method using GAPDH as the housekeeper gene [31].

**Table 1:** RT-PCR primer sequences designed by PRIMER 3, version 0.4.0

Mouse TLR4	Forward: 5‘-CTC TGC CTT CAC TAC AGA GAC-3’ Reverse: 5’-TGG ATG ATG TTG GCA GCA ATG-3’
Mouse MD2	Forward: 5‘- GTC CGA TGG TCT TCC TGG CGA GT-3’ Reverse: 5’ GCT TCT CAG ATT CAG TCA ATA TGG G-3’
Mouse CD14	Forward: 5‘- GTC AGG AAC TCT GGC TTT GC-3’ Reverse: 5’ GGC TTT TAC CCA CTG AAC CA-3’
Mouse MyD88	Forward: 5‘- GGA GCC AGA TTC TCT GAT GC-3’ Reverse: 5’ TGT CCC AAA GGA AAC ACA CA-3’
Mouse IL-6	Forward: 5‘- AGT TGC CTT CTT GGG ACT GA-3’ Reverse: 5’ TCC ACG ATT TCC CAG AGA AC-3’
Mouse IL-6 Receptor	Forward: 5‘- TGA ATG ATG ACC CCA GGC AC-3’ Reverse: 5’ ACA CCC ATC CGC TCT CTA CT-3’
Mouse CXCL2	Forward: 5‘- AAG TTT GCC TTG ACC CTG AA-3’ Reverse: 5’ AGG CAC ATC AGG TAC GAT CC-3’
Mouse CXCR1	Forward: 5‘- GGG TGA AGC CAC AAC AGA TT-3’ Reverse: 5’ GCA GAC CAG CAT AGT GAG CA-3’
Mouse CXCR2	Forward: 5‘- GCA GAG GAT GGC CTA GTC AG-3’ Reverse: 5’ TCC ACC TAC TCC CAT TCC TG-3’
Mouse GAPDH (housekeeper)	Forward: 5‘- CCT CGT CCC GTA GAC AAA ATG-3’ Reverse: 5’ TCT CCA CTT TGC CAC TGC AA-3’

### ELISAs of Human Cell Culture and Mouse Colonic Explant Supernatants

Human IL-8 production was measured in cell culture supernatant using an ELISA kit (Abcam, Cambridge, United Kingdom) following the manufacturer’s instructions. Mouse IL-6 production was measured in intestinal explant culture supernatant using an ELISA kit (Invitrogen, Massachusetts, United States) following the manufacturer's instructions. Absorbance was measured at 450 nm using a Synergy™ Mx Monochromator-Based Multi-Mode Microplate Reader (BioTek). Absorbance output was calculated and converted into protein concentration using a standard curve from the ELISA kit (IL-8: 1000 – 31.25 pg/mL; IL-6: 500 – 4 pg/mL) and the GraphPad Prism Software version 9.0 (GraphPad® Software, USA).

### Statistical Analysis

Data was graphed and analysed using the GrahPad Prism Software 9.0 (GraphPad^®^ Software, San Diego, USA). A Kruskal–Wallis test with Dunn’s multiple comparisons test was performed on non-parametric data to compare between the treatment groups. An ordinary one-way ANOVA with Tukey’s multiple comparisons test was performed on parametric data to compare between the treatment groups. Any data point that had a higher value than 3 times the standard deviation from the mean was excluded. *P*-values of < 0.05 were considered statistically significant.

## RESULTS

### Effect of TAK-242 and IAXO-102 Treatment on Cell Viability (Cytostasis)

**Fig. 1:**
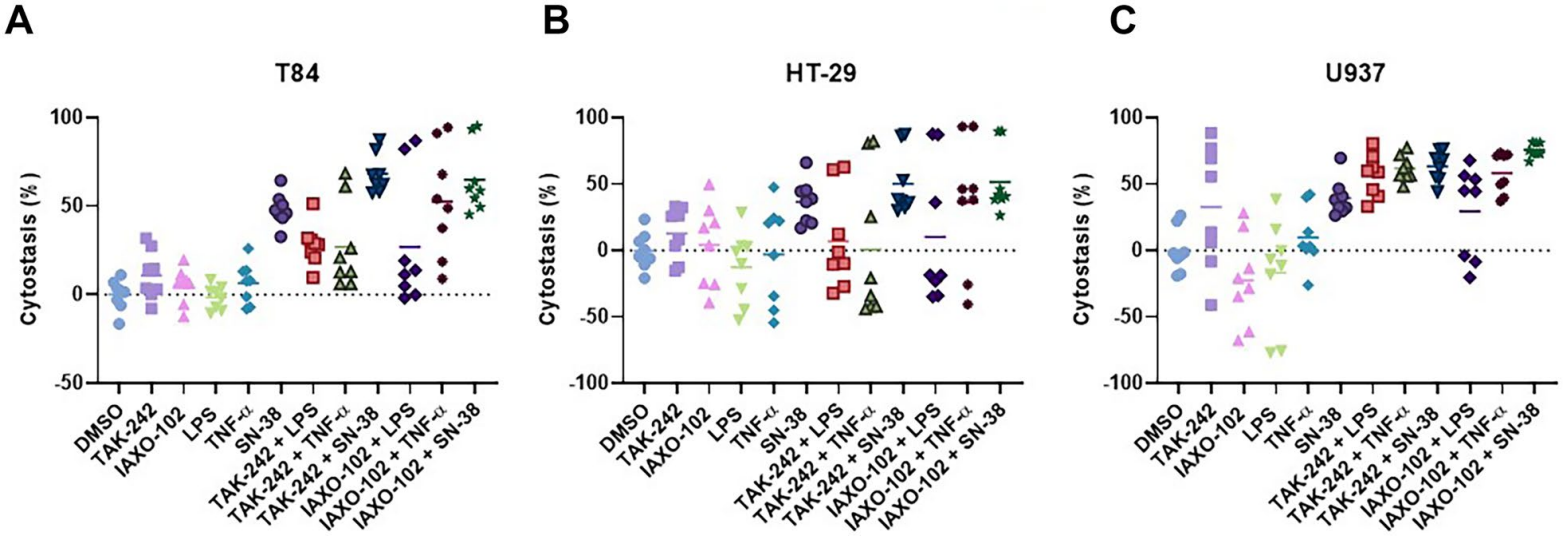
Effect of TLR4 antagonism on cell cytostasis following LPS (100 µg/mL), TNF-α (5 µg/mL), and SN-38 treatment (T84 and HT-29: 1 µM; U937: 1 nM) in (A) T84, (B) HT-29, and (C) U937 cell lines. 0.1% DMSO was used as the vehicle. **A** T84: DMSO *vs.* SN-38 (*P* < 0.0001), DMSO *vs.* TAK-242 + SN-38 (*P* < 0.0001), DMSO *vs.* IAXO-102 + TNF-α (*P* < 0.0001), DMSO *vs.* IAXO-102 + SN-38 (*P* < 0.0001), TAK-242 *vs.* SN-38 (*P* < 0.01), TAK-242 *vs.* TAK-242 + SN-38 (*P* < 0.0001), TAK-242 *vs.* IAXO-102 + TNF-α (*P* < 0.01), TAK-242 *vs.* IAXO-102 + SN-38 (*P* < 0.0001), IAXO-102 *vs.* SN-38 (*P* < 0.001), IAXO-102 *vs.* TAK-242 + SN-38 (*P* < 0.0001), IAXO-102 *vs.* IAXO-102 + TNF-α (*P* < 0.001), IAXO-102 *vs.* IAXO-102 + SN-38 (*P* < 0.0001), LPS *vs.* SN-38 (*P* < 0.0001), LPS *vs.* TAK-242 + SN-38 (*P* < 0.0001), LPS *vs.* IAXO-102 + TNF-α (*P* < 0.0001), LPS *vs.* IAXO-102 + SN-38 (*P* < 0.0001), TNF-α *vs.* SN-38 (*P* < 0.01), TNF-α *vs.* TAK-242 + SN-38 (*P* < 0.0001), TNF-α *vs.* IAXO-102 + TNF-α (*P* < 0.001), TNF-α *vs.* IAXO-102 + SN-38 (*P* < 0.0001), TAK-242 + LPS *vs.* TAK-242 + SN-38 (*P* < 0.01), TAK-242 + LPS *vs.* IAXO-102 + SN-38 (*P* < 0.0001), TAK-242 + TNF-α *vs.* TAK-242 + SN-38 (*P* < 0.01), TAK-242 + TNF-α *vs.* IAXO-102 + SN-38 (*P* < 0.0001), TAK-242 + SN-38 *vs.* IAXO-102 + LPS (*P* < 0.01), IAXO-102 + LPS *vs.* IAXO-102 + SN-38 (*P* < 0.0001). **B** HT-29: LPS *vs.* TAK-242 + SN-38 (*P* < 0.05), LPS *vs.* IAXO-102 + SN-38 (*P* < 0.05). **C** U937: DMSO *vs.* TAK-242 + LPS (*P* < 0.01), DMSO *vs.* TAK-242 + TNF-α (*P* < 0.001), DMSO *vs.* TAK-242 + SN-38 (*P* < 0.001), DMSO *vs.* IAXO-102 + TNF-α (*P* < 0.001), DMSO *vs.* IAXO-102 + SN-38 (*P* < 0.0001), TAK-242 *vs.* IAXO-102 (*P* < 0.01), TAK-242 *vs.* LPS (*P* < 0.05), IAXO-102 *vs.* SN-38 (*P* < 0.001), IAXO-102 *vs.* TAK-242 + LPS (*P* < 0.0001), IAXO-102 *vs.* TAK-242 + TNF-α (*P* < 0.0001), IAXO-102 *vs.* TAK-242 + SN-38 (*P* < 0.0001), IAXO-102 *vs.* IAXO-102 + LPS (*P* < 0.01), IAXO-102 *vs.* IAXO-102 + TNF-α (*P* < 0.0001), IAXO-102 *vs.* IAXO-102 + SN-38 (*P* < 0.0001), LPS *vs.* SN-38 (*P* < 0.01), LPS *vs.* TAK-242 + LPS (*P* < 0.0001), LPS *vs.* TAK-242 + TNF-α (*P* < 0.0001), LPS *vs.* TAK-242 + SN-38 (*P* < 0.0001), LPS *vs.* IAXO-102 + LPS (*P* < 0.05), LPS *vs.* IAXO-102 + TNF-α (*P* < 0.0001), LPS *vs.* IAXO-102 + SN-38 (*P* < 0.0001), TNF-α *vs.* TAK-242 + LPS (*P* < 0.05), TNF-α *vs.* TAK-242 + TNF-α (*P* < 0.01), TNF-α *vs.* TAK-242 + SN-38 (*P* < 0.01), TNF-α *vs.* IAXO-102 + TNF-α (*P* < 0.05), TNF-α *vs.* IAXO-102 + SN-38 (*P* < 0.001), IAXO-102 + LPS *vs.* IAXO-102 + SN-38 (*P* < 0.05). Data are presented as mean (*n* = 8 per group).

Since a hallmark feature of IM leading to inflammation is cell loss, we measured cytostasis in three different cell lines: T84, HT-29, and U937. The TLR4 antagonists, TAK-242 (10 µM) and IAXO-102 (10 µM), alone did not cause cytostasis (*P* > 0.05, Fig. 1). However, they also did not provide protection against cytostasis following treatments with LPS (100 µg/mL), TNF-α (5 µg/mL), and SN-38 treatment (T84 and HT-29: 1 µM; U937: 1 nM) in any of the cell lines (*P* > 0.05, Fig. 1).

**Fig. 2:**
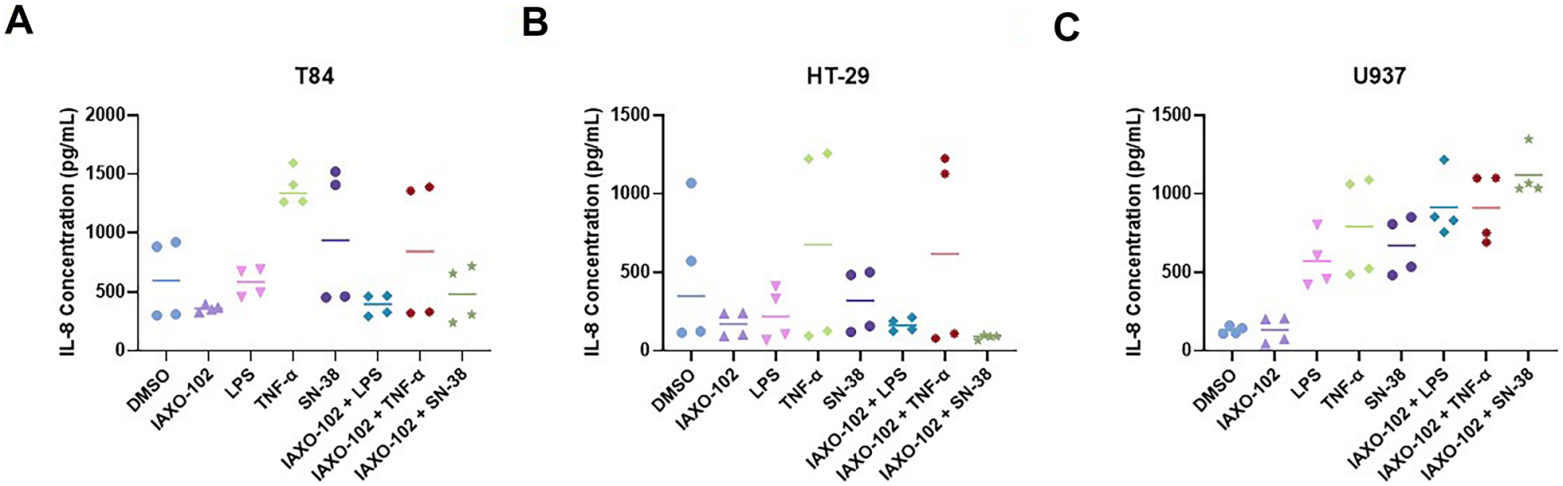
Effect of the TLR4 antagonist IAXO-102 (10 µM) on suppression of IL-8 secretion following LPS (100 µg/mL), TNF-α (5 µg/mL), and SN-38 treatment (T84 and HT-29: 1 µM; U937: 1 nM) in **A** T84, **B** HT-29, and **C** U937 cell lines. 0.1% DMSO was used as the vehicle. There was no significant difference in IL-8 secretion between the treated and vehicle groups in the T84 and HT-29 cell lines (*P* > 0.05). **C** U937: DMSO *vs.* TNF-α (*P* < 0.01), DMSO *vs.* SN-38 (*P* < 0.05), DMSO *vs.* IAXO-102 + LPS (*P* < 0.001), DMSO *vs.* IAXO-102 + TNF-α (*P* < 0.001), DMSO *vs.* IAXO-102 + SN-38 (*P* < 0.0001), IAXO-102 *vs.* TNF-α (*P* < 0.01), IAXO-102 *vs.* SN-38 (*P* < 0.05), IAXO-102 *vs.* IAXO-102 + LPS (*P* < 0.001), IAXO-102 *vs.* IAXO-102 + TNF-α (*P* < 0.001), IAXO-102 *vs.* IAXO-102 + SN-38 (*P* < 0.0001), LPS *vs.* IAXO-102 + SN-38 (*P* < 0.01), SN-38 *vs.* IAXO-102 + SN-38 (*P* < 0.05). Data are presented as median (T84 and HT-29) and mean (U937) (*n* = 4 per group).

### Effect of IAXO-102 Treatment on IL-8 Production

In human models of inflammation, IL-8 is a key downstream cytokine released following TLR4 activation. As such, IL-8 secretion was tested in intestinal and immune cell lines. All 3 cell lines produced an IL-8 secretory response following treatment (Fig. 2). Due to the similarity observed in the cytostasis results of all 3 cell lines, we proceeded to focus only on IAXO-102 due to its novel aspect compared to TAK-242 which is already a popular research compound. We found the TLR4 antagonist IAXO-102 (10 µM), alone did not cause an IL-8 secretory response (*P* > 0.05, Fig. 2). However, they also did not suppress any IL-8 secretory responses following treatments with LPS (100 µg/mL), TNF-α (5 µg/mL), and SN-38 treatment (T84 and HT-29: 1 µM; U937: 1 nM) in any of the cell lines (*P* > 0.05, Fig. 2).

### Histopathological Analysis and Immunohistochemistry (IHC) Assessment of Cellular Markers of Apoptosis and Proliferation of Mouse Colonic Explants

**Fig. 3:**
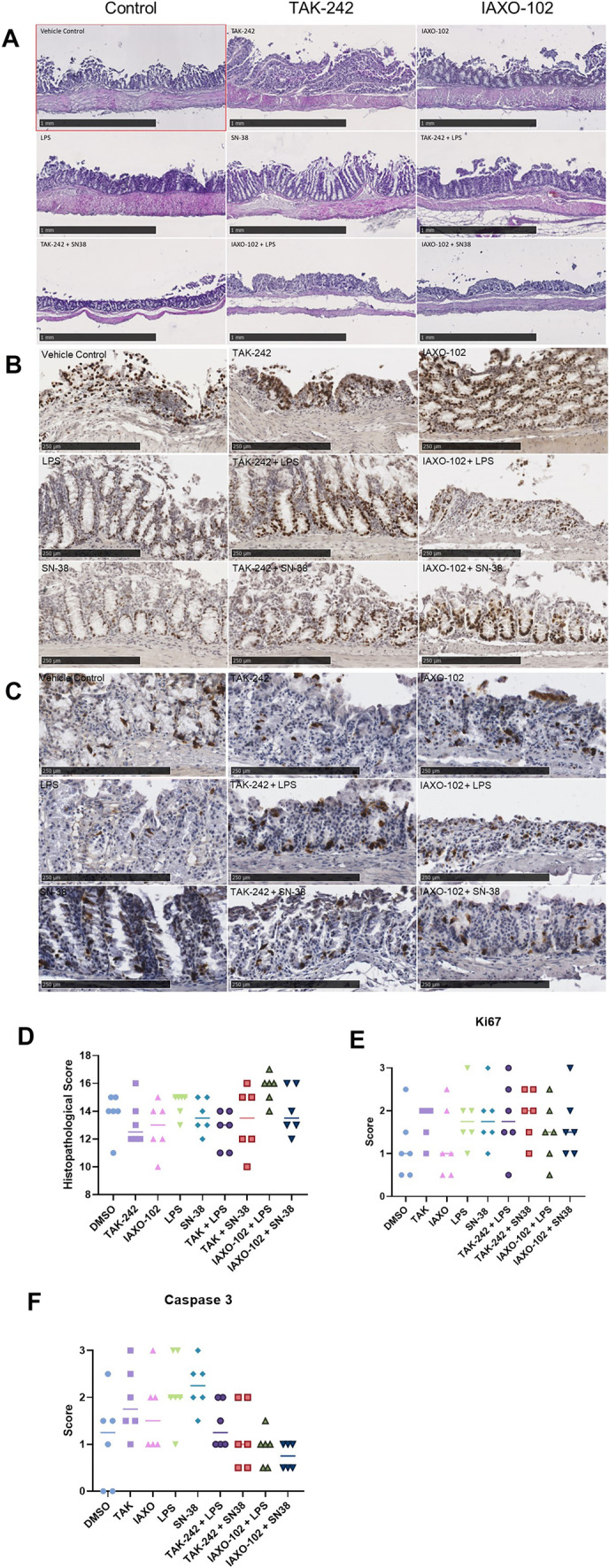
**A** Representative H&E stained colonic explants following treatments. 0.2% DMSO was used as the vehicle. **D** Histopathological analysis of the H&E images from multiple mice. 0.2% DMSO was used as the vehicle. No significant differences in histopathological scores in the colonic explants between the treated and vehicle tissue was observed (*P* > 0.05). Data are presented as median (*n* = 6 per group). **B** Representative images of colonic explants following treatments stained with Ki67 (brown staining). **C** Representative images of colonic explants following treatments stained with Caspase 3 (brown staining). **E** Analysis and scoring of the colonic explants stained with Ki67. No significant differences in scores in the colonic explants between the treated and vehicle tissue was observed (*P* > 0.05). **F** Analysis and scoring of the colonic explants stained with Caspase 3. DMSO *vs.* TAK-242 (*P* < 0.01), DMSO *vs.* LPS (*P* < 0.0001), DMSO *vs.* SN-38 (*P* < 0.0001), TAK-242 *vs.* TAK-242 + SN-38 (*P* < 0.01), TAK-242 *vs.* IAXO-102 + LPS (*P* < 0.0001), TAK-242 *vs.* IAXO-102 + SN-38 (*P* < 0.0001), IAXO-102 *vs.* IAXO-102 *vs.* LPS (*P* < 0.01), IAXO-102 *vs.* IAXO-102 *vs.* SN-38 (*P* < 0.001), LPS *vs.* TAK-242 + LPS (*P* < 0.01), LPS *vs.* TAK-242 + SN38 (*P* < 0.0001), LPS *vs.* IAXO-102 + LPS (*P* < 0.0001), LPS *vs.* IAXO-102 + SN38 (*P* < 0.0001), SN-38 *vs.* TAK-242 + LPS (*P* < 0.01), SN-38 *vs.* TAK-242 + SN38 (*P* < 0.0001), SN-38 *vs.* IAXO-102 + LPS (*P* < 0.0001), SN-38 *vs.* IAXO-102 + SN38 (*P* < 0.0001). 0.2% DMSO was used as the vehicle. Data presented as median (Ki67) and mean (Caspase 3), *n* = 6 per group.

The ability to model inflammation in single cell lines is limited, thus we adapted a colonic explant model to further examine TLR4 signalling in mucositis development. No histological changes were observed in the mouse colon explants after treatment with DMSO (0.2%), TAK-242 (10 µM), IAXO-102 (10 µM), LPS (100 µg/mL), and SN-38 (2 µM) (Fig. 3A). All sections showed infiltration of neutrophils and disruption of the epithelial layer with no distinguishable differences observed between colon tissue treated with the TLR4 antagonists, TAK-242 and IAXO-102, or pro-inflammatory mediators (Fig. 3A). This is supported by no differences in the quantitative histopathological scores in the colonic explants following any treatments (*P* > 0.05, Fig. 3D). 

To follow up on the cell viability assay, Ki67 IHC staining was used to determine cell proliferation while Caspase 3 IHC staining was used to determine presence of apoptotic cells in the explants treated with DMSO (0.2%), TAK-242 (10 µM), IAXO-102 (10 µM), LPS (100 µg/mL), and SN-38 (2 µM) (Fig. 3B, C, respectively). All sections displayed widely distributed staining of Ki67 with no distinguishable differences observed between colon tissue treated with the TLR4 antagonists, TAK-242 and IAXO-102, or pro-inflammatory mediators (Fig. 3B). This is supported by no differences in the quantitative scores in the colonic explants following any treatments (*P* > 0.05, Fig. 3E). As for caspase 3, apoptosis was observed to be decreased in the explant tissues treated with both the antagonist and inflammatory mediator compared to explants tissues treated with either DMSO only, antagonist only, or inflammatory mediator only (Fig. 3F).

### Secretion of Pro-inflammatory Cytokine IL-6 from Mouse Colonic Explants

**Fig. 4:**
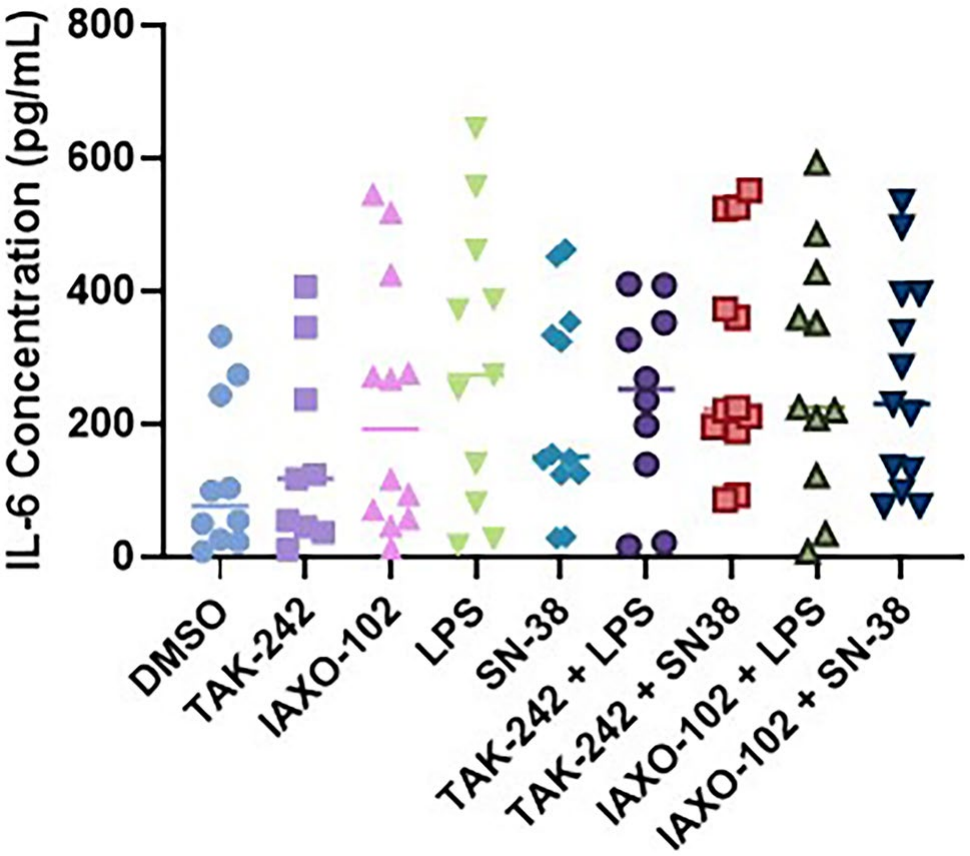
IL-6 secretion from mouse colonic explant supernatant following various treatments: DMSO (0.2%), TAK-242 (10 µM), IAXO-102 (10 µM), LPS (100 µg/mL), and SN-38 (2 µM). TAK-242 and IAXO-102 did not significantly inhibit IL-6 concentration after treatment with inflammatory mediators and TLR4 agonists LPS and SN-38 (*P* > 0.05). Data are presented as median (*n* = 13 per group).

Histological visualisation is not sufficient to evaluate release of pro-inflammatory signals that may contribute to mucositis development. As such, we measured secretion of the key inflammatory cytokine linked to intestinal tissue inflammation in mucositis, IL-6. Inflammatory mediators and TLR4 agonists LPS (100 µg/mL) and SN-38 (2 µM) did not significantly increase the IL-6 secretion in the explant media (*P* > 0.05, Fig. 4). In addition, the TLR4 antagonists TAK-242 (10 µM) and IAXO-102 (10 µM) did not cause IL-6 secretion alone or alter the secretion of IL-6 following treatments (*P* > 0.05, Fig. 4).

### Effect of TAK-242 and IAXO-102 Treatment on Gene Expression in Colonic Mouse Explants

**Fig. 5:**
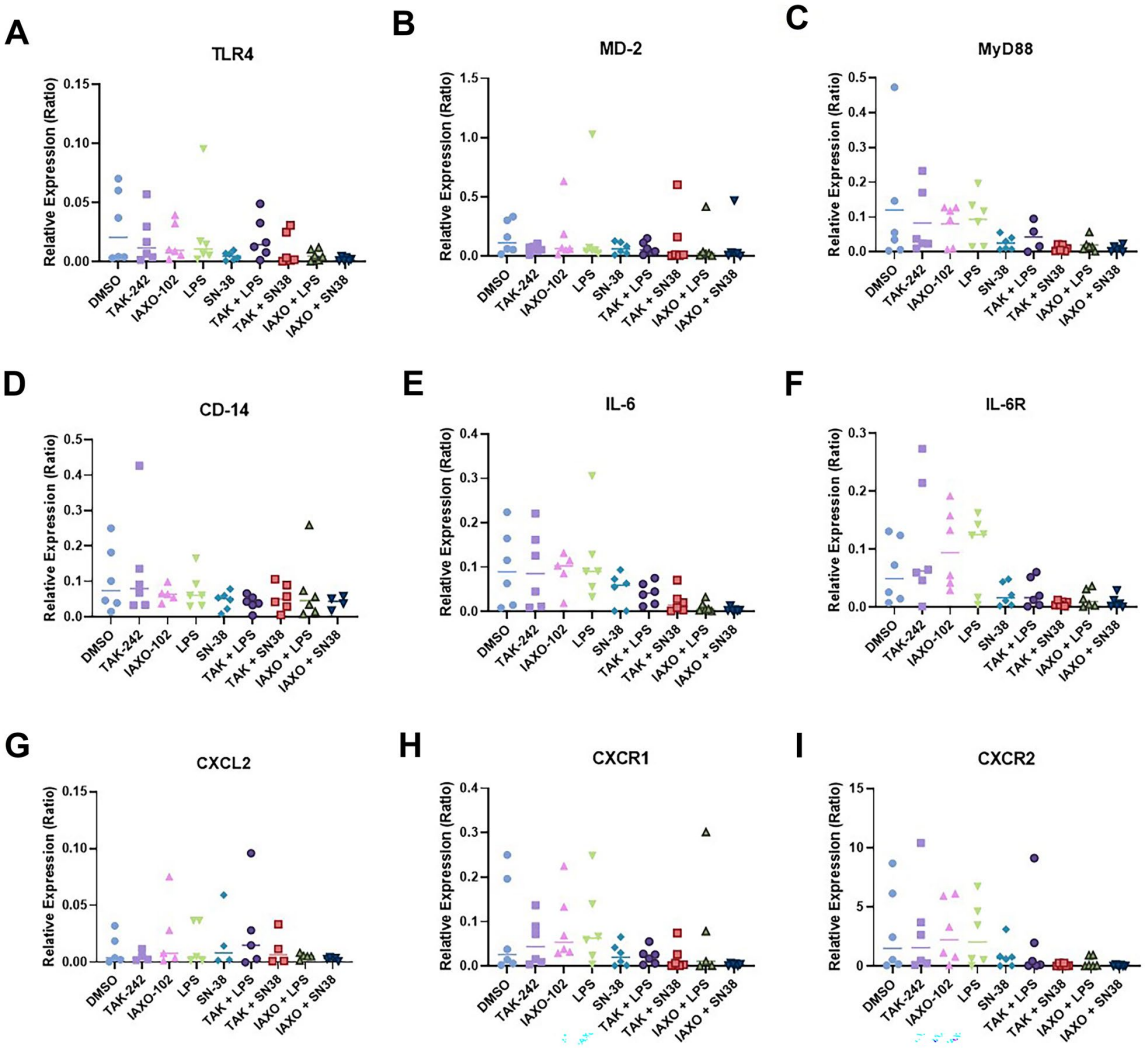
Relative gene expression of **A** TLR4, **B** MD2, **C** MyD88, **D** CD14, **E** IL-6, **F** IL-6R, **G** CXCL2, **H** CXCR1, and **I** CXCR2 from mouse colonic explants following various treatments: TAK-242 (10 µM), IAXO-102 (10 µM), LPS (100 µg/mL), and SN-38 (2 µM). 0.2% DMSO was used as the vehicle. No significant upregulation of the genes was observed in the tissue treated with LPS and SN-38 (*P* > 0.05). No significant downregulation of the genes was observed in tissue pre-treated with TAK-242 and IAXO-102 (*P* > 0.05). Data are presented as median (*n* = 6 per group).

We decided to look at the levels of expression of genes associated with the TLR4/MD2 downstream signalling pathway. LPS (100 µg/mL) and SN-38 (2 µM) stimulation did not result in higher transcription levels of the associated genes TLR4, MD2, MyD88, CD14, IL-6, IL-6R, CXCL2, CXCR1, and CXCR2 (*P* > 0.05, Fig. 5). In addition, there was no change observed in gene expression when the explants had been pre-treated with the TLR4 antagonists TAK-242 (10 µM) and IAXO-102 (10 µM) (*P* > 0.05, Fig. 5).

## DISCUSSION

IM is defined as the inflammation of the mucosa of the intestinal tract and is a side-effect of high dose chemotherapy. Patients who develop IM will often suffer from its side-effects such as pain, nausea, and diarrhoea. These side-effects can be so debilitating that the life-saving treatments patients with cancer undergo need to be stopped, which considerably effects their survival. However, TLR4 signalling has been strongly implicated in the development of intestinal mucositis. Evidence from *in* *vitro* studies using human cells and *in vivo* studies using animal models supports the hypothesis that the activation of TLR4 is related to the pathogenesis of intestinal inflammation [19, 32]. Studies have also shown evidence that when TLR4 is inhibited, there was a decrease in inflammatory infiltrate and protection against damage. For example, a study by Ungaro et al. showed that mice with intestinal inflammation that were pre-treated with TAK-242 had a decreased disease activity index (DAI) score and IL-6 secretion (*P* < 0.05 *vs.* disease only) [12]. While a study by Fort et al. found that the DAI and histological score was significantly decreased (*P* < 0.05 *vs.* vehicle) in mice pre-treated with a synthetic TLR4 antagonist CRX-526 (50 µg) [33].

Therefore, the TLR4 activation and signalling provides a strong target for pharmacological intervention. This study therefore aimed to determine how TLR4 inhibition protects against intestinal mucositis using preclinical models of inflammation. It was the first time the specific TLR4 antagonists, TAK-242 and IAXO-102, have been investigated in such models. We observed that these TLR4 antagonists did not display any toxic side effects in the preclinical models of inflammation.

In our *in vitro* model, the cell lines T84, HT-29, and U937 had different relative expression levels of TLR4, but this did not impact their response to TLR4 antagonists. The concentration of TLR4 antagonists used in this study was determined by previous studies using the same compounds [25, 34]. All three cell lines displayed a relatively similar reaction as seen in our results of cell cytostasis and secretion of inflammatory mediator IL-8. However, the TLR4 antagonists were not able to prevent any inflammation or cell death induced by LPS, TNF-α, and SN-38. Although it was observed that the TLR4 antagonists did not protect against SN-38 in our cell cytostasis results, this is consistent with studies showing that TLR4 is required for healing in colitis [12].

When we observed the results from the human IL-8 ELISA, it was observed that all 3 cell lines secreted the inflammatory cytokine after being treated with LPS, TNF-α, and SN-38. However, when the cell lines with LPS, TNF-α, and SN-38 were pre-treated with IAXO-102, there was no decrease in IL-8 observed. Compared to a study by Huggins et al. [25] stimulated human umbilical cord vein endothelial cells (HUVEC) with LPS (100 ng/mL) with and without pre-treatment of IAXO-102 (1 and 10 µM), with IAXO-102 (10 µM) inhibiting the secretion of IL-8 (*P* < 0.01 against LPS only) [25].

One possible reason for the contradictory *in vitro* results is that the intestinal epithelium, i.e. the T84 and HT-29 cells, must remain mute to the presence of commensal flora and bacterial pathogens to avoid a constant need to defend the intestinal environment against invading pathogens. The colonic epithelial cell types would also be exposed heavily to LPS as they are the main protective barrier between the lumen and the lamina propria. Due to the constant exposure to LPS, these cell types may limit their response to LPS and bacterial pathogens by downregulating the TLR4/MD2 expression [32]. Another limitation is that immortalised cells do not mimic a human intestinal tract as they are derived from human tumour cells. As such, TLR4 signalling may not reflect the healthy intestine and primary cell lines may need to be considered for future work.

This limitation was why an explant model was used in the present study, to better mimic a healthy intestinal tract system. However, similar results were observed in the explant model of intestinal inflammation as were observed in the *in vitro* cell lines; no significant protection against inflammation was observed in colonic explants treated with TLR4 antagonists and the inflammatory mediators or TLR4 agonists. This indicates that inhibition of TLR4 was not able to suppress the inflammatory response. The unexpected lack of response highlights the limitations of *ex vivo* explant models in cytotoxic studies and that IL secretion did not reflect the damage in colon architecture and morphology caused by cytotoxics in the colon.

Possible explanations for these results maybe that it was too late to inhibit TLR4 to see a significant reaction. Due to the nature of explants, inflammatory signals would have been released and cells would undergo apoptosis when the intestine was removed from the mouse. When the tissue was divided into the wells, the media would have been saturated with pro-inflammatory mediators as seen in the increase in IL-6 secretion in our colonic explant model. When the TLR4 antagonist was finally added to the tissue, a difference in cytokine secretions would be unidentifiable. Which may be why TLR4 knockout models of mice are so effective at preventing mucositis. A similar reason can be used to explain the variability seen in the gene expression of the explant tissue. Whereby due to the degradation of the colonic explants, the DNA extracted from the tissue may have been compromised.

However, previous studies using intestinal explants have been quite successful. For example, a study by Guabiraba et al. [26] reported no increase in the pro-inflammatory cytokine IL-33 in their colonic explants, but when SN-38 was added, there was a significant increase in the IL-33 levels (*P* < 0.001 *vs.* vehicle) [26]. Mouse intestinal explants are a popular model to use in a variety of studies [35, 36]. However, none of these studies has provided any histology on the tissue. As such, our study extends knowledge in the field and limitation of explant tissue models. As such, whole animal studies are preferable.

In conclusion, the results demonstrated no protective capabilities of TLR4 antagonism against the *in vitro* and *ex vivo* intestinal inflammation models. Although T84 and HT-29 cells mimic the colonic epithelial cell phenotype in many regards, these cell lines cannot replicate all stages of colonic epithelial cell differentiation and lack key microbiomes that may be important for TLR4 signalling [37]. The explant models would also need to be refined to prevent the tissue from degrading rapidly in order to provide more consistent results or more viable, long-term models such as organoids, or other co-culture systems considered. Further investigation on the specific binding sites of these TLR4 antagonists should also be considered. Therefore, our data must be interpreted with the considerations of the inherent limitations of these systems. However, we were able to fill a knowledge gap in the explant model with our histology findings.
